# Screening, optimization, and ADMET evaluation of HCJ007 for pancreatic cancer treatment through active learning and dynamics simulation

**DOI:** 10.3389/fchem.2024.1482758

**Published:** 2024-11-25

**Authors:** YunYun Xu, Qiang Wang, GaoQiang Xu, YouJian Xu, YiPing Mou

**Affiliations:** ^1^ General Surgery, Cancer Center, Department of Gastrointestinal and Pancreatic Surgery, Zhejiang Provincial People’s Hospital (Affiliated People’s Hospital), Hangzhou Medical College, Hangzhou, Zhejiang, China; ^2^ General Surgery, Tiantai People’s Hospital, Taizhou, Zhejiang, China

**Keywords:** marine natural products, screening of SQLE inhibitors, active learning model, ADMET analysis, molecular dynamics simulations, molecular modification

## Abstract

In this study, we leveraged a sophisticated active learning model to enhance virtual screening for SQLE inhibitors. The model’s improved predictive accuracy identified compounds with significant advantages in binding affinity and thermodynamic stability. Detailed analyses, including molecular dynamics simulations and ADMET profiling, were conducted, particularly focusing on compounds CMNPD11566 and its derivative HCJ007. CMNPD11566 showed stable interactions with SQLE, while HCJ007 exhibited improved binding stability and more frequent interactions with key residues, indicating enhanced dynamic adaptability and overall binding effectiveness. ADMET data comparison highlighted HCJ007s superior profile in terms of lower toxicity and better drug-likeness. Our findings suggest HCJ007 as a promising candidate for SQLE inhibition, with significant improvements over CMNPD11566 in various pharmacokinetic and safety parameters. The study underscores the efficacy of computational models in drug discovery and the importance of comprehensive preclinical evaluations.

## 1 Introduction

The relentless challenge posed by pancreatic cancer, with its high mortality rate and limited treatment options, underscores the urgent need for innovative therapeutic strategies (Halbrook et al., 2023). The emerging role of cholesterol metabolism in cancer progression has opened new avenues for research (Xiao et al., 2023), particularly focusing on squalene epoxidase (SQLE). This enzyme, pivotal in cholesterol metabolism, is notably overexpressed in pancreatic cancer, presenting an intriguing therapeutic target (You et al., 2022). SQLE’s significant upregulation in pancreatic cancer tissues correlates with poor prognosis, as observed in recent studies (Xu et al., 2023). This enzyme not only accelerates cell proliferation and cell cycle progression but also inhibits apoptosis, both *in vitro* and *in vivo*. SQLE’s mechanistic action is twofold: it alleviates endoplasmic reticulum stress and activates the lipid raft-mediated Src/PI3K/Akt signaling pathway, thus propelling pancreatic cancer growth. The efficacy of SQLE inhibitors in reducing pancreatic cancer cell proliferation and impeding xenograft tumor growth further underscores SQLE’s potential as a therapeutic target.

In addition to marine-derived compounds, several synthetic and semi-synthetic SQLE inhibitors have been explored for their potential efficacy. Notable examples include Naftifine, Terbinafine, Butenafine, Tolnaftate, Ellagic acid, and Epigallocatechin gallate (EGCG). Naftifine, Terbinafine, and Butenafine are well-established antifungal agents that inhibit SQLE, reducing ergosterol synthesis and disrupting fungal cell membrane integrity. Tolnaftate, another antifungal, also targets SQLE and has been shown to effectively treat skin infections. Ellagic acid and EGCG, though primarily known for their antioxidant and anti-inflammatory properties, have demonstrated potential in inhibiting cholesterol metabolism and related pathways, making them promising candidates for SQLE inhibition in cancer therapeutics. These compounds highlight the diverse chemical scaffolds and mechanisms that can be harnessed in the development of effective SQLE inhibitors.

Marine natural products (MNPs) serve as a crucial source for developing novel anticancer agents (Khalifa et al., 2019). Due to their unique chemical structures and diverse biological activities, these compounds are viewed as a promising avenue for novel cancer therapies. For instance, iodinated carrageenan, isolated from red algae, has been employed in treating respiratory infections caused by rhinoviruses (Frediansyah, 2021). Trabectedin, extracted from the Caribbean sea squirt Ecteinascidia turbinate, is used for treating ovarian cancer and soft tissue sarcoma (Carter and Keam, 2010). Brentuximab vedotin, derived from the marine mollusk Dolabella auricularia, is utilized in the treatment of Hodgkin lymphoma and large cell lymphoma (Donato et al., 2018). Another example is Ziconotide, isolated from the venom of the marine snail Conus magnus, which is an effective analgesic, a thousand times more potent than morphine, and hence approved for pain management in HIV and cancer patients (Banik and Engle, 2020). Overall, the exploration of marine natural products offers a rich resource and potential new pathways for developing novel anticancer drugs, especially in unraveling the complex and varied mechanisms of cancer diseases.

With advancements in technology and deeper understanding of marine biodiversity, more unique marine natural products with therapeutic potential are expected to be discovered (McConnell et al., 1994). The integration of computational biology in drug discovery, particularly in screening marine natural products, marks a significant shift in identifying new therapeutic agents (Abduljalil et al., 2023). Utilizing advanced computational tools like active learning algorithms, molecular dynamics simulations, and diverse scoring systems, we can efficiently explore vast libraries of marine natural products (Tran et al., 2021). This approach allows for the rapid and precise identification of potential SQLE inhibitors, predicting their effectiveness and safety profiles with greater accuracy.

This study aims to leverage the chemical diversity of marine natural products to identify novel SQLE inhibitors for treating pancreatic cancer. By adopting an integrative computational strategy, we aim to methodically screen a marine natural products library, uncovering promising SQLE inhibitors. This endeavor is not only pivotal in advancing pancreatic cancer therapeutics but also showcases the vast potential of marine natural products in drug discovery, particularly when augmented by computational biology techniques.

## 2 Materials and methods

### 2.1 Protein preparation

In our quest to discover inhibitors targeting SQLE, we adopted structure-based computational techniques, focusing on the SQLE crystal structure (PDB ID: 6C6N) complexed with Cmpd-4” (Padyana et al., 2019). This particular structure was chosen to serve as the receptor model. The preparation of the receptor-ligand complex was carried out using Schrödinger’s Protein Preparation Wizard, which involved a series of meticulous steps. These included the addition of missing hydrogen atoms, adjustment of metal ion states, bond order determination in HET groups, assessment and optimization of ligand protonation states along with their energy implications, tuning protonation states of histidine residues, correcting any misplaced heavy atoms, refining the hydrogen bonding network within the protein, and executing a restrained minimization to ensure structural integrity. The identified binding site within the 3D structure of the receptor, where Cmpd-4″ interacts, was designated as the focal point for screening potential ligands. Consequently, a grid corresponding to this target site was generated to facilitate the screening process.

### 2.2 Active learning based virtual screening

Active Learning Glide will generate a receptor grid from a prepared protein, prepare the Comprehensive Marine Natural Products Database (https://cmnpd.org/), and dock a subset of these ligands using Glide SP(Friesner et al., 2004). Active Learning workflows train a machine learning (ML) model on physics-based data, such as FEP+(Wang et al., 2015) predicted affinities or Glide docking scores, iteratively sampled from a full library using Schrödinger’s deep-learning powered QSAR platform, DeepAutoQSAR (https://www.schrodinger.com/science-articles/benchmark-study-deepautoqsar-chemprop-and-deeppurpose-admet-subset-therapeutic-data). Three iterative training rounds were set. After all the ligands have been screened using the last model, a selection of the top ligands will then be docked using Glide SP. The results of the docking were then quantified based on the consensus of docking scores and Prime MM-GBSA energy (Mittal et al., 2021; 2020).

### 2.3 Binding pose metadynamics

To verify the stability of binding poses of the selected ligands from molecular dynamics (MD) simulations in both binding sites, a sequence of metadynamics MD simulations was executed, each lasting for 10 ns, on various docked poses and MD-established stable protein-ligand complexes (Clark et al., 2016; Fusani et al., 2020; Purushotham et al., 2022). The chosen collective variable (CV) was the root mean square deviation (RMSD) of the ligand’s heavy atoms from their initial positions, computed post alignment of the binding sites to mitigate any drift. The parameters for the hill’s height and width in the metadynamics simulations were set at 0.05 kcal/mol and 0.02 Å, respectively. The system underwent solvation in a box with a 10 Å buffer, followed by a series of minimization steps, gradually elevating the system’s temperature to 300 K and alleviating any initial structural stresses or contacts. The evaluation of stability was based on monitoring the RMSD fluctuations of the ligand throughout the simulation (termed as PoseScore) and quantifying the average duration of critical contacts between the ligand and protein residues (referred to as PersScore).

### 2.4 ADMET screening and drug-likeness predictions

To ensure favorable ADMET (absorption, distribution, metabolism, excretion, and toxicity) properties and non-toxicity of potential drug candidates, the study employed ADMETLAB 2.0 (https://admetmesh.scbdd.com/) (Xiong et al., 2021) for prediction. Adherence to medicinal chemistry guidelines, such as the Lipinski (Lipinski et al., 2001), Pfizer (Hughes et al., 2008), GSK(Gleeson, 2008), and Golden Triangle rules (Johnson et al., 2009), was pivotal in identifying compounds with optimal ADMET attributes.

### 2.5 Molecular dynamic simulation

The Desmond software from Schrödinger LLC was employed for conducting molecular dynamics (MD) simulations over a period of 1,000 ns. The TIP3P water model, representing a three-point intermolecular interaction potential, was chosen for these simulations. The simulations were set up within an orthorhombic box, maintaining a constant temperature of 300 K and pressure of 1 atm, while utilizing the OPLS 2005 force field (Shivakumar et al., 2010). To achieve neutrality in the models and mimic physiological conditions, counter ions were added, and a 0.15 M sodium chloride solution was used to simulate ligand-binding status in physiological environment (Rasheed et al., 2021). Prior to the commencement of the simulations, the models underwent a relaxation phase, and during the simulations, data was recorded and stored every 100 ps for later analysis.

## 3 Results

### 3.1 Application of active learning model in the screening of potential SQLE inhibitors

**FIGURE 1:**
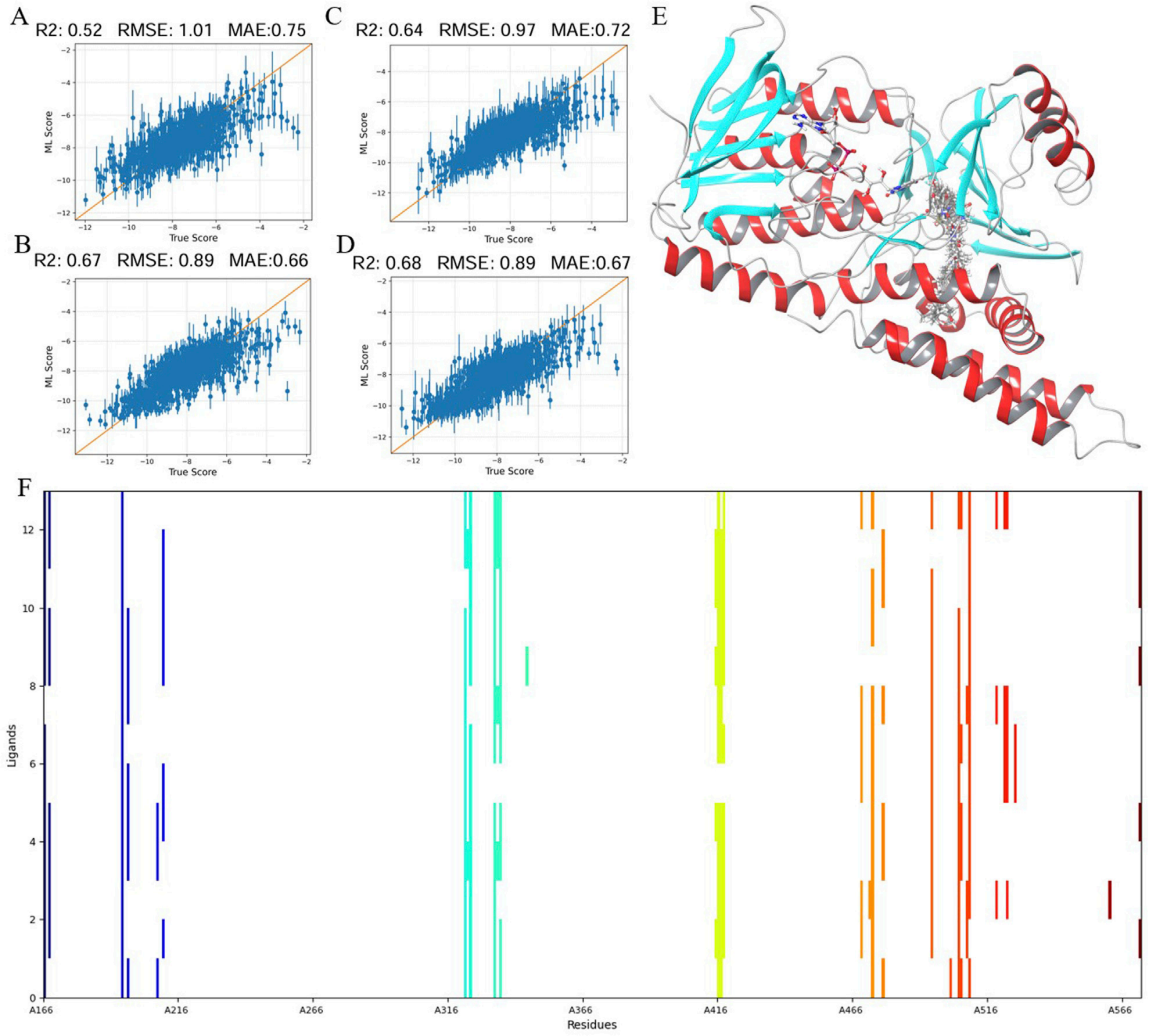
Application of active learning model in the screening of potentional SQLE inhibitors: **(A–D)** Training results of the active learning model at different iterative stages. **(E)** Enrichment effect of SQLE target compounds after active learning combined with multidimensional scoring. **(F)** Interaction fingerprints of 13 selected compounds with SQLE.

Using an active learning model, we have improved the precision of virtual screening to identify SQLE inhibitors, as shown in Figure 1. The model’s iterations, visualized in Figures 1A–D, show an increase in predictive accuracy, with *R* ^2^ values rising from 0.52 to 0.68. This improvement demonstrates the model’s enhanced ability to identify genuine inhibitors from a complex chemical library. Figure 1E shows the spatial distribution of the selected inhibitors within the SQLE binding pocket, highlighting their structural compatibility with the active site. Figure 1F provides an interaction fingerprint analysis, describing the key contacts between inhibitors and important amino acid residues of SQLE, which is crucial for further optimizing these molecules.

In Table 1, the selected compounds consistently show a state penalty of zero, indicating alignment with the target enzyme’s active site and minimal off-target interactions. Their docking scores range from −10.036 to −11.644, surpassing known inhibitors, which suggests strong binding affinity. The ligand strain energy remains below 10 kcal/mol, indicating that these compounds can adopt a stable conformation in the enzyme’s pocket. The MMGBSA binding free energy values are strongly negative, ranging from −60.47 to −73.52 kcal/mol, indicating stable interactions, especially for compound 18,775, which shows the most favorable binding energy. These values compare favorably to established inhibitors (−49.18 to −60.66 kcal/mol), positioning the screened compounds as potentially more effective inhibitors.

**TABLE 1:** Detailed scoring of selected compounds.

CMNPD	MMGBSA dG bind	Lig strain energy	State penalty	Docking score
18,775	−73.52	4.051	0	−10.437
8663	−72.62	8.891	0	−10.774
8801	−71.32	9.497	0	−10.186
9,511	−71.01	7.035	0	−10.326
8904	−70.96	6.991	0	−10.14
31,347	−69.95	7.476	0	−10.152
1792	−69.31	9.173	0	−10.038
11,566	−67.54	9.752	0	−10.075
8875	−63.03	7.986	0	−10.083
30,653	−62.76	7.047	0	−10.275
18,677	−61.72	7	0	−11.644
18,987	−61.69	7.318	0	−10.843
8819	−60.47	9.694	0	−10.036

Overall, the selected compounds, with their high docking scores, low strain energy, and favorable MMGBSA binding energies, show great promise as effective SQLE inhibitors. They offer potential improvements over current pharmacological agents and represent a significant advancement in virtual screening results, bringing us closer to discovering new therapeutic options.

### 3.2 Analysis of compound interactions and scoring in BPMD simulations

Binding Pose Metadynamics (BPMD) is an advanced technique that uses automated and enhanced sampling based on metadynamics to explore the dynamic binding poses of ligands. This approach is effective in distinguishing the correct binding pose of ligands from less accurate poses that can result from conventional docking methods. Using BPMD, we analyzed the stability of thirteen compounds over ten simulations, each lasting 10 nanoseconds. The collective variable root mean square deviation (CV RMSD) profiles focused on compounds that maintained stability within a fluctuation threshold of 2 Å. Scoring metrics such as PoseScore, PersScore, and CompScore were used to refine the selection. A lower PoseScore indicates better stability, while a higher PersScore shows sustained hydrogen bond presence. CompScore, combining these factors, identifies the most stable complexes.

**FIGURE 2:**
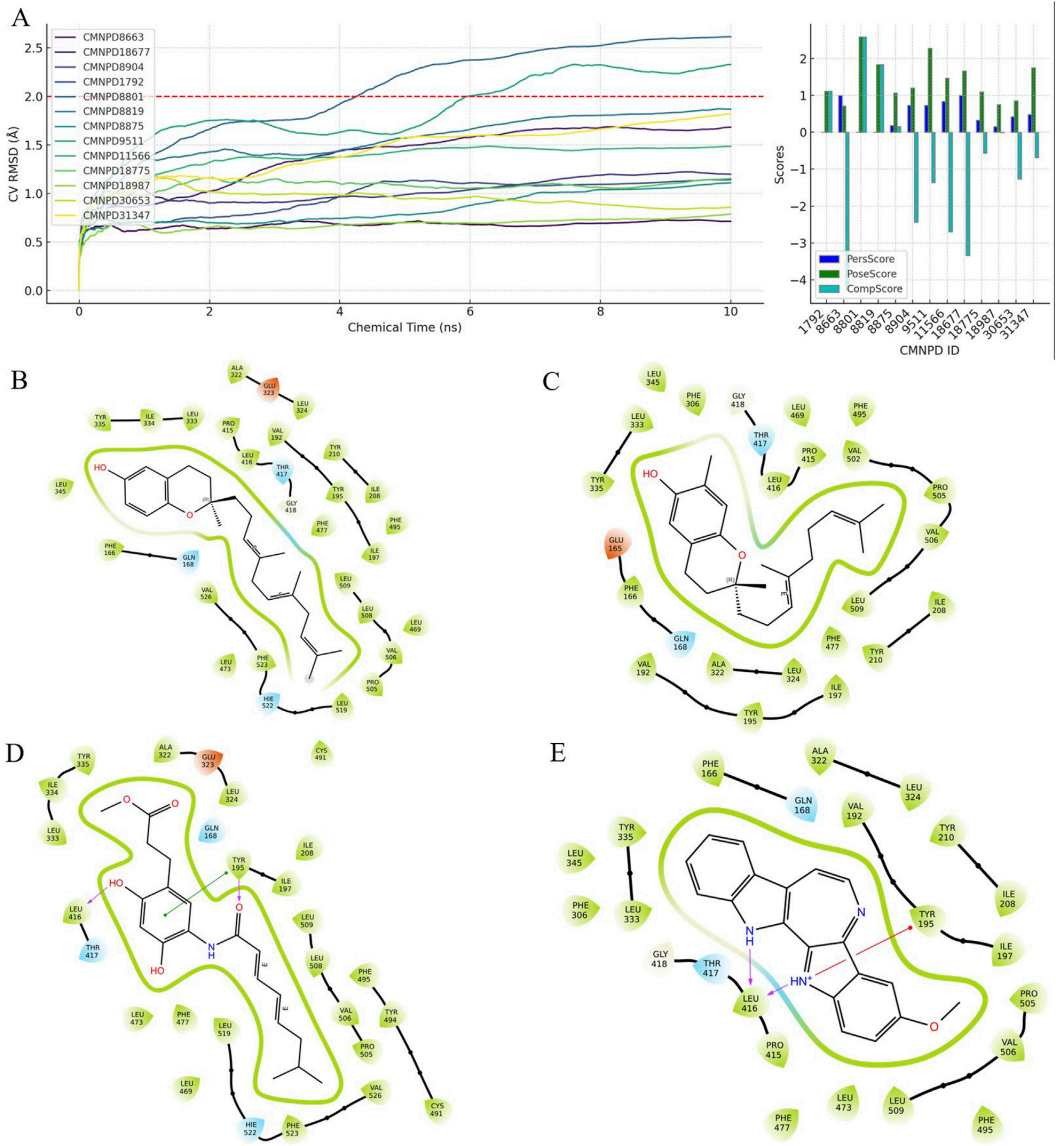
Analysis of compound interactions and scoring in BPMD simulations: **(A)** Left: Dynamic changes in CV RMSD over time for 13 compounds during 10 ns BPMD simulations repeated 10 times. Right: Three different scoring scenarios for the 13 compounds. **(B–E)** Four compounds selected through BPMD screening: CMNPD8663, CMNPD8904, CMNPD11566, and CMNPD18677, respectively.

Among the analyzed compounds, CMNPD8663, CMNPD8904, CMNPD11566, and CMNPD18677 demonstrated optimal stability, as shown in Figure 2A. CMNPD8663 (Figure 2B) mainly depends on hydrophobic interactions within the SQLE receptor’s pocket, which serve as the key to its binding affinity, even without specific hydrogen bonds. CMNPD8904 (Figure 2C) also relies on hydrophobic interactions, with potential Pi-Pi stacking involving Tyr-195 contributing to binding specificity. CMNPD11566 (Figure 2D) shows a more complex interaction profile, combining hydrophobic contacts and hydrogen bonds with Tyr-195 and Leu-416, enhancing stability and specificity. CMNPD18677 (Figure 2E) exhibits a diverse binding mode, involving extensive hydrophobic interactions, a salt bridge with Tyr-195, and hydrogen bonds with Leu-416 and Thr-417, indicating high affinity and selectivity.

These findings highlight the diverse types of interactions between the ligands and the SQLE receptor, emphasizing the combination of hydrophobic forces, hydrogen bonds, and salt bridges. Notably, CMNPD11566 and CMNPD18677 showed advanced binding properties, suggesting their potential as effective SQLE inhibitors.

### 3.3 Evaluation of physicochemical properties and drug-likeness criteria for selected compounds

ADMET analysis plays a crucial role in drug development by providing key insights into how a compound behaves in the body. This includes absorption, distribution, metabolism, excretion, and toxicity—factors that collectively determine a compound’s potential as a drug.

**FIGURE 3:**
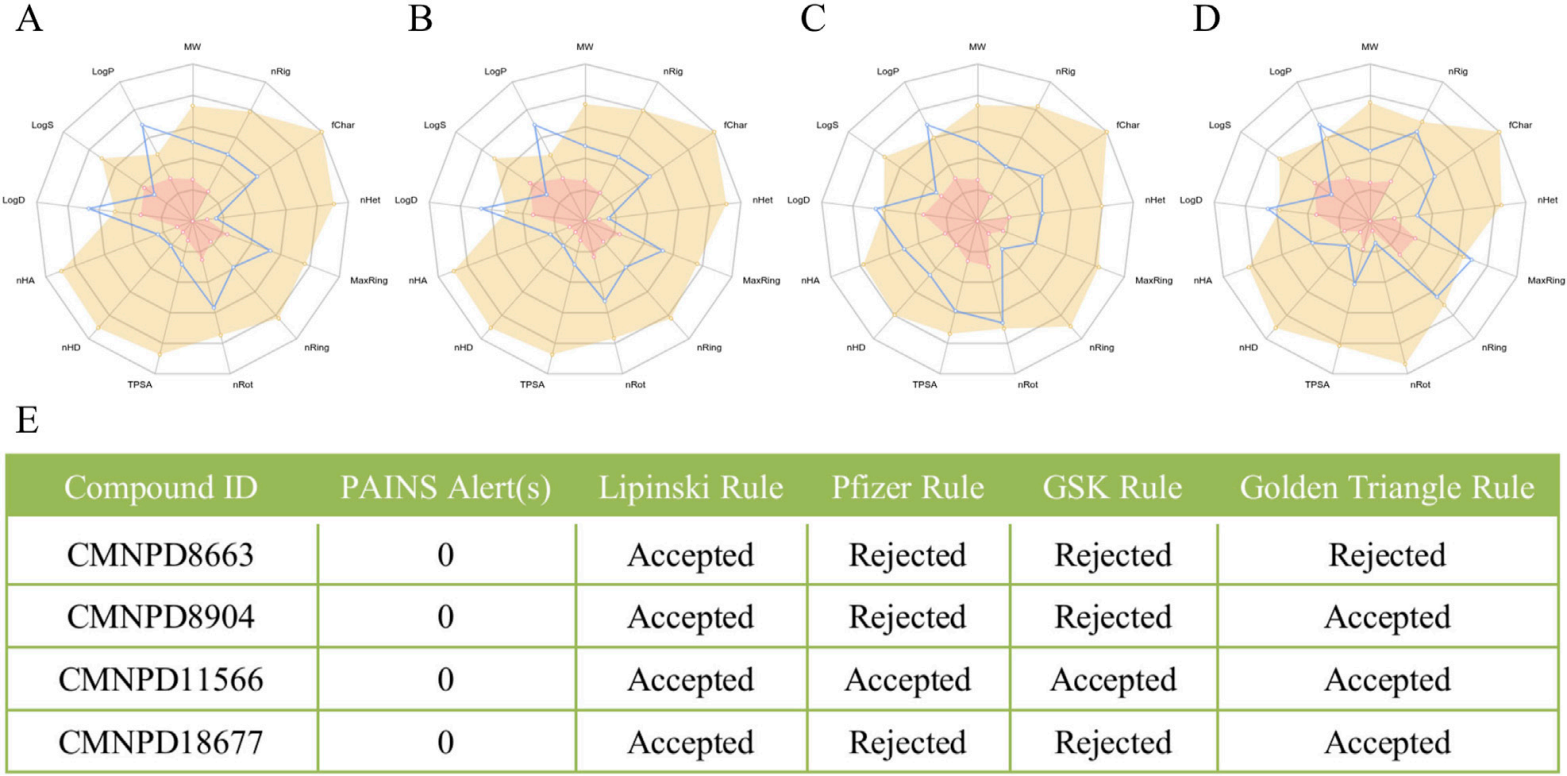
Evaluation of physicochemical properties and drug-likeness criteria for selected compounds: **(A–D)** Radar charts of physicochemical properties for four compounds: CMNPD8663, CMNPD8904, CMNPD11566, and CMNPD18677, respectively. **(E)** Compliance with Lipinski’s rule of five and the number of PAINS (Pan-Assay Interference Compounds) alerts for the four compounds.

Regarding physicochemical properties (Figures 3A–D), the compounds CMNPD8663, CMNPD8904, CMNPD11566, and CMNPD18677 each show distinct profiles. CMNPD8663 and CMNPD8904 exhibit moderate properties that are favorable for permeability and distribution. CMNPD11566 has an especially favorable profile, with a lower molecular weight and smaller polar surface area, indicating potential for good oral bioavailability. CMNPD18677, with its broader range of properties, suggests potential for more complex interactions in biological systems.

Figure 3E summarizes the drug-likeness of these compounds. CMNPD8904 follows Lipinski’s Rule, suggesting it could be orally available, while CMNPD11566 stands out by meeting all applied criteria (including Lipinski, Pfizer, GSK, and the Golden Triangle), making it the most promising candidate in terms of drug-likeness.

Focusing on CMNPD11566, its ADMET characteristics (Supplementary Table S1) show that, despite some challenges in Caco-2 permeability, MDCK permeability suggests effective passive diffusion. The compound has a high plasma protein binding percentage, meaning that most of it will be bound in circulation, with only a small fraction available for pharmacological effects. It also interacts with several Cytochrome P450 enzymes, which could lead to drug-drug interactions that need careful monitoring.

Clearance and half-life data indicate that CMNPD11566 is eliminated at a moderate rate and has a short duration in the body, which will affect dosing frequency. Toxicity predictions highlight areas of concern, such as potential genotoxicity and skin sensitization, which require careful evaluation.

In conclusion, while CMNPD11566 has favorable physicochemical properties and an excellent drug-likeness profile, its comprehensive ADMET analysis suggests the need for a careful approach in its development. Detailed preclinical studies will be critical to address the challenges in its ADMET profile and fully realize its therapeutic potential.

### 3.4 Molecular dynamics simulation and biophysical analysis of CMNPD11566 and its derivative HCJ007 in interaction with SQLE

**FIGURE 4:**
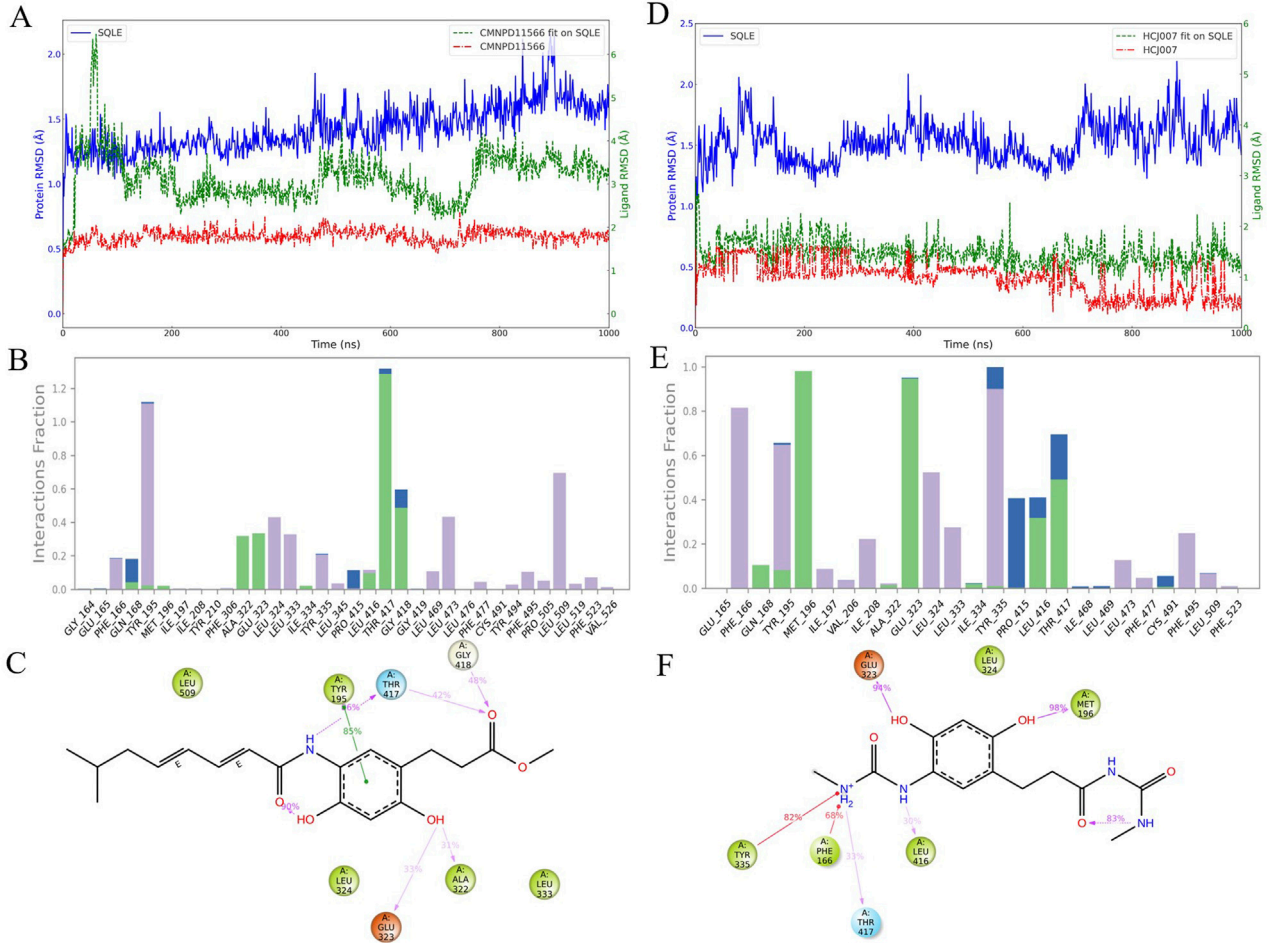
Molecular dynamics simulation analysis of CMNPD11566 and its derivative HCJ007: **(A–C)** Simulations for CMNPD11566: **(A)** Three types of RMSD for CMNPD11566. **(B)** Interaction statistics of CMNPD11566. **(C)** 2D interaction diagram of CMNPD11566 with residues having interaction frequency over 30%. **(D–F)** Simulations for the derivative HCJ007: **(D)** Three types of RMSD for HCJ007. **(E)** Interaction statistics of HCJ007. **(F)** 2D interaction diagram of HCJ007 with residues having interaction frequency over 30%.

Molecular dynamics simulations are crucial for understanding the structure-activity relationships of drug candidates and their interactions with target proteins. In the 1,000 ns molecular dynamics simulation of CMNPD11566 (Figures 4A–C), we observed several potential limitations. Figure 4A shows the RMSD trajectories of CMNPD11566, with fluctuations between 1 and 3 Å. While this suggests relatively stable interactions with the SQLE enzyme, there is still room for improvement. Figure 4B indicates that CMNPD11566 interacts frequently with residues like Tyr-195 and Thr-417 but has low interaction frequencies with residues such as Met-196 and Ile-197, implying possible instability in its structure-activity relationship with SQLE. Figure 4C further shows that the alkyl chain of CMNPD11566 does not form stable interactions with residues in the binding pocket, contributing to low interaction frequencies.

Due to the mobility of CMNPD11566s alkyl chain, which may cause binding stability issues, we decided to modify its structure. Based on an analysis of CMNPD11566s interaction patterns, we designed a new compound, HCJ007, to address these binding issues while retaining effective interactions with key residues.

Simulations of HCJ007 (Figures 4D–F) showed significant improvements in its dynamic properties. Figure 4D shows that HCJ007 has a more stable RMSD, indicating better binding conformation stability. Figure 4E reveals that HCJ007 has increased interaction frequencies with key residues and fewer low-frequency interaction sites. Figure 4F shows detailed interaction patterns of HCJ007, demonstrating stronger and more consistent binding interactions compared to CMNPD11566, suggesting its potential for more effective binding and therapeutic action.

In developing HCJ007 as a potent therapeutic against SQLE, we performed a series of detailed biophysical analyses to understand its interaction dynamics and molecular stability. Throughout its modification process, HCJ007 consistently met the four key drug-likeness rules and showed no PAINS (Pan-Assay Interference Compounds) alerts, as noted in Supplementary Table S2. These insights guided the design of HCJ007 to enhance its specificity and efficacy.

**FIGURE 5:**
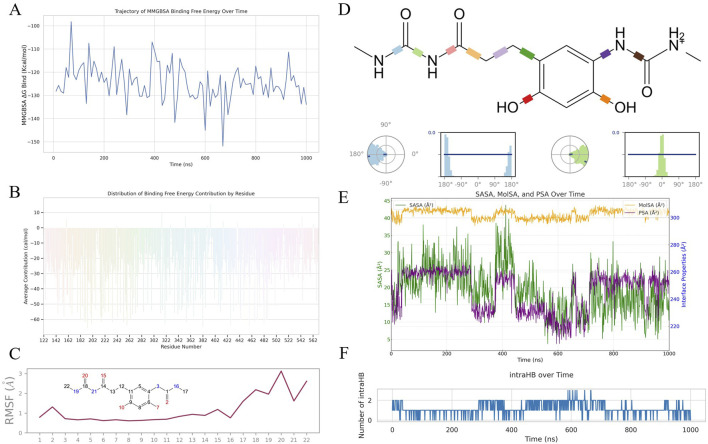
Comprehensive biophysical analysis of HCJ007 in interaction with SQLE:**(A)** MMGBSA binding free energy between HCJ007 and SQLE. **(B)** Contribution of individual residues to the HCJ007-SQLE complex stability. **(C)** RMSF analysis of HCJ007 **(D)** Torsion angle analysis of HCJ007. **(E)** Interface properties analysis of HCJ007, including SASA, MolSA, and PSA. **(F)** Intra-molecular hydrogen bonding patterns in HCJ007.

Figure 5A shows the evolving affinity between HCJ007 and SQLE through MMGBSA binding free energy values. The deepening negative trend, averaging at −124.48 kcal/mol and reaching as low as −151.82 kcal/mol, highlights HCJ007s potential as a high-affinity inhibitor, demonstrating strong engagement with SQLE. Figure 5B further explores the contributions of individual residues, showing that residues like Asn-193 and Asn-382 play crucial roles in stabilizing the HCJ007-SQLE complex, providing guidance for drug optimization.

At the atomic level, Figure 5C provides insights into HCJ007s internal dynamics. The increased RMSF from atom 16 indicates retained flexibility within the alkyl chain, which is important for adaptable binding. Figure 5D’s torsion angle analysis shows minimal fluctuations, supporting the structural integrity of the alkyl chain, ensuring proper alignment during binding.

Figures 5E, F focus on molecular stability and interaction dynamics. Figure 5E highlights interface properties like SASA, MolSA, and PSA, which modulate the hydrophobic-hydrophilic balance. Figure 5F shows a consistent pattern of intra-molecular hydrogen bonding, which is a key indicator of molecular stability and functional integrity. The occasional transient spikes indicate HCJ007s ability to adapt dynamically to structural shifts within the complex. These properties are closely connected, driving the binding dynamics and stability of the HCJ007-SQLE complex.

Overall, these analyses provide a detailed picture of HCJ007 as a well-optimized compound for efficacy. Each molecular and atomic interaction is designed to synergize with SQLE, positioning HCJ007 as a promising therapeutic candidate.

## 4 Discussion

Leveraging the diverse chemical landscape of marine-derived compounds, this study has identified promising squalene epoxidase (SQLE) inhibitors, advancing the search for effective pancreatic cancer treatments. The application of an active learning model has refined virtual screening processes, pinpointing compounds with high binding affinity and requisite stability within the SQLE active site. This innovative approach has enabled the discovery of compounds that not only exhibit high theoretical efficacy but also hold potential for significant advancements in SQLE inhibition.

Building on this foundation, our comprehensive analysis using multiple scoring criteria for the thirteen compounds has shown significant advantages in various aspects. Notably, the docking scores of all selected compounds exceeded −10, with CMNPD18677 achieving −11.644, indicating exceptionally strong binding affinity with SQLE (Meli et al., 2022). This is further corroborated by the state penalty of zero for all compounds, emphasizing their high specificity in binding to SQLE and reducing the likelihood of non-specific interactions (Friesner et al., 2006).

Additionally, ligand strain energy is a crucial metric for assessing how a ligand’s energy changes when binding to a target protein (Tong and Zhao, 2021). Ideally, lower ligand strain energy means that the ligand can more naturally adapt to the protein’s binding pocket, reducing the energy loss caused by conformational changes and increasing binding affinity. In our screening results, most selected compounds exhibited ligand strain energy values below 10 kcal/mol, significantly lower than known inhibitors like Butenafine (17.45 kcal/mol), Naftifine (9.666 kcal/mol), and Terbinafine (8.458 kcal/mol). For instance, compound 18,775 had a strain energy of only 4.051 kcal/mol, much lower than Butenafine. This suggests that these compounds may bind more easily to SQLE, as they require less conformational change. These findings highlight the effectiveness of using an active learning model to select marine natural products as SQLE inhibitors. Compounds with low strain energy are theoretically more suitable as drug candidates, as they demand less energy to bind to their target proteins, potentially enhancing their efficacy and stability within the body.

Most impressively, the MMGBSA dG Bind scores reveal that all selected compounds have significantly lower scores compared to known inhibitors. Particularly remarkable is CMNPD18775, which scored −73.52 kcal/mol, far surpassing the scores of Butenafine, Naftifine, and Terbinafine. This indicates that the complexes formed with SQLE are likely to be more thermodynamically stable and potentially more effective as inhibitors (Pattar et al., 2020). This finding underscores the effectiveness of the active learning model in efficiently identifying potent and stable SQLE inhibitors from a vast repository of marine-derived compounds.

Transitioning to the dynamic characteristics, CMNPD11566 exhibited limitations in its interaction dynamics with SQLE, as seen in the molecular dynamics simulations. Its RMSD trajectories showed fluctuations within the range of 1–3 Å, suggesting areas for optimization despite relatively stable interactions (Kumar et al., 2023). The interaction frequency analysis revealed high-frequency interactions with residues like Tyr-195 and Thr-417 but lower interaction frequencies with others, indicating potential instability and suboptimal interaction with the enzyme’s binding pocket. Tyr-195 was identified as a crucial residue in the SQLE enzyme. This residue plays a significant role in inhibitor binding, as evidenced by the fact that both NB-598 (Horie et al., 1990) and Cmpd-4″ establish a hydrogen bond with Tyr-195, which is the only specific and directional interaction these compounds have with SQLE (Padyana et al., 2019). This interaction is consistent across all known SQLE inhibitors and explains the required presence of the tertiary amine motif in these inhibitors. The interaction with conserved Tyr-195 is critical for the binding and efficacy of SQLE inhibitors.

In contrast, HCJ007 displayed markedly improved dynamic characteristics. Its more stable RMSD, enhanced binding conformation stability, and increased interaction frequencies with key residues, as seen in subsequent simulations, highlight its potential for more effective binding and therapeutic action. This comparison underscores the effectiveness of the structural modifications made to HCJ007, resulting in improved interaction dynamics essential for successful SQLE inhibition.

Finally, the ADMET data comparison reveals that HCJ007 offers significant improvements over CMNPD11566 in various parameters, such as lower BBB penetration and PPB, suggesting reduced CNS exposure and improved systemic circulation of the active drug (Tong et al., 2022). Despite a higher clearance rate, HCJ007s comparable half-life to CMNPD11566 suggests similar durations in the body. Additionally, HCJ007 shows significantly reduced toxicity in AMES test results and skin sensitization, lower risks of hERG cardiac toxicity and DILI, and potentially better drug-like properties as indicated by its higher QED score.

The clinical implications of these findings are significant. The identification of HCJ007 as a potent SQLE inhibitor with superior binding stability, safety, and pharmacokinetic profile suggests its potential use as a novel therapeutic agent for pancreatic cancer. Given that SQLE is overexpressed in pancreatic cancer and plays a role in tumor progression through cholesterol metabolism, targeting this enzyme with HCJ007 could lead to reduced tumor growth and improved patient outcomes. The enhanced ADMET profile of HCJ007, including reduced toxicity and better systemic circulation, positions it as a promising candidate for further clinical development. Importantly, the reduction in BBB penetration implies minimized central nervous system side effects, which is crucial for improving the quality of life in cancer patients undergoing treatment. Future clinical trials will be essential to determine the therapeutic efficacy, optimal dosage, and safety of HCJ007 in human subjects. The transition from computational and *in vitro* findings to clinical settings will require a well-designed approach that includes dose-escalation studies, pharmacokinetic assessments, and long-term toxicity evaluations. These steps are vital to ensure that HCJ007 can be effectively translated from preclinical promise to a viable treatment option for patients.

Based on these findings, the study propels us into new realms of research and clinical development, particularly focusing on the potential of HCJ007 as a SQLE inhibitor. With its superior dynamic characteristics and significant ADMET advantages over CMNPD11566, particularly in toxicity, safety, and drug-likeness, HCJ007 stands as a strong candidate for further exploration. This research lays a foundation for innovative approaches in drug discovery, combining computational modeling with empirical data to refine drug efficacy and safety profiles. The next steps involve validating these results through *in vitro* and *in vivo* studies, aiming towards eventual clinical trials. Such progression towards translational research emphasizes the crucial role of comprehensive preclinical evaluations, ensuring that compounds like HCJ007 are not only effective in laboratory settings but also well-suited for real-world therapeutic applications.

While our study presents promising findings based on computational modeling, we acknowledge that the absence of experimental validation is a limitation of this work. The results obtained through active learning, docking, and molecular dynamics simulations have not yet been tested in biological assays, such as *in vitro* enzymatic inhibition or *in vivo* animal studies. Experimental validation, including these biological assays, is crucial for confirming the efficacy and safety of the identified compounds, particularly HCJ007.

To address this limitation, our future research plans include conducting *in vitro* studies to assess the inhibitory potential of HCJ007 on squalene epoxidase activity in cell cultures. Additionally, we aim to perform *in vivo* pharmacokinetic and toxicity studies to establish HCJ007s suitability for clinical applications. We also recognize that *in silico* approaches, while powerful for initial screenings, require experimental backing to fully understand the compound’s efficacy, toxicity, and potential off-target effects in a physiological context.

## 5 Conclusion

This study marks a significant advancement in identifying potent squalene epoxidase (SQLE) inhibitors for pancreatic cancer treatment. Utilizing an active learning model, we identified compounds, notably HCJ007, with superior binding affinity, stability, and ADMET properties compared to known inhibitors. HCJ007s enhanced dynamic characteristics and reduced toxicity profiles position it as a promising therapeutic candidate. These findings pave the way for further validation through *in vitro* and *in vivo* studies, potentially leading to clinical trials and offering new avenues in cancer treatment.

## Data Availability

The original contributions presented in the study are included in the article/supplementary material, further inquiries can be directed to the corresponding author.

## References

[B1] Abduljalil J. M. Elfiky A. A. Sayed E.-S. T. A. AlKhazindar M. M. (2023). Computational identification of drug-like marine natural products as potential RNA polymerase inhibitors against Nipah virus. Comput. Biol. Chem. 104, 107850. 10.1016/j.compbiolchem.2023.107850 36907056

[B2] Banik R. K. Engle M. P. (2020). Ziconotide for management of cancer pain refractory to pharmacotherapy: an update. Pain Med. 21, 3253–3259. 10.1093/pm/pnaa251 32940675

[B3] Carter N. J. Keam S. J. (2010). Trabectedin: a review of its use in soft tissue sarcoma and ovarian cancer. Drugs 70, 335–376. 10.2165/11202860-000000000-00000 20166769

[B4] Clark A. J. Tiwary P. Borrelli K. Feng S. Miller E. B. Abel R. (2016). Prediction of protein-ligand binding poses via a combination of induced fit docking and metadynamics simulations. J. Chem. Theory Comput. 12, 2990–2998. 10.1021/acs.jctc.6b00201 27145262

[B5] Donato E. M. Fernández-Zarzoso M. Hueso J. A. de la Rubia J. (2018). Brentuximab vedotin in Hodgkin lymphoma and anaplastic large-cell lymphoma: an evidence-based review. Onco Targets Ther. 11, 4583–4590. 10.2147/ott.s141053 30122950 PMC6084082

[B6] Frediansyah A. (2021). The antiviral activity of iota-kappa-and lambda-carrageenan against COVID-19: a critical review. Clin. Epidemiol. Glob. Health 12, 100826. 10.1016/j.cegh.2021.100826 34222718 PMC8240443

[B7] Friesner R. A. Banks J. L. Murphy R. B. Halgren T. A. Klicic J. J. Mainz D. T. (2004). Glide: a new approach for rapid, accurate docking and scoring. 1. Method and assessment of docking accuracy. J. Med. Chem. 47, 1739–1749. 10.1021/jm0306430 15027865

[B8] Friesner R. A. Murphy R. B. Repasky M. P. Frye L. L. Greenwood J. R. Halgren T. A. (2006). Extra precision glide: docking and scoring incorporating a model of hydrophobic enclosure for protein-ligand complexes. J. Med. Chem. 49, 6177–6196. 10.1021/jm051256o 17034125

[B9] Fusani L. Palmer D. S. Somers D. O. Wall I. D. (2020). Exploring ligand stability in protein crystal structures using binding pose metadynamics. J. Chem. Inf. Model 60, 1528–1539. 10.1021/acs.jcim.9b00843 31910338 PMC7145342

[B10] Gleeson M. P. (2008). Generation of a set of simple, interpretable ADMET rules of thumb. J. Med. Chem. 51, 817–834. 10.1021/jm701122q 18232648

[B11] Halbrook C. J. Lyssiotis C. A. Pasca di Magliano M. Maitra A. (2023). Pancreatic cancer: advances and challenges. Cell 186, 1729–1754. 10.1016/j.cell.2023.02.014 37059070 PMC10182830

[B12] Horie M. Tsuchiya Y. Hayashi M. Iida Y. Iwasawa Y. Nagata Y. (1990). NB-598: a potent competitive inhibitor of squalene epoxidase. J. Biol. Chem. 265, 18075–18078. 10.1016/s0021-9258(17)44716-8 2211682

[B13] Hughes J. D. Blagg J. Price D. A. Bailey S. Decrescenzo G. A. Devraj R. V. (2008). Physiochemical drug properties associated with *in vivo* toxicological outcomes. Bioorg Med. Chem. Lett. 18, 4872–4875. 10.1016/j.bmcl.2008.07.071 18691886

[B14] Johnson T. W. Dress K. R. Edwards M. (2009). Using the Golden Triangle to optimize clearance and oral absorption. Bioorg Med. Chem. Lett. 19, 5560–5564. 10.1016/j.bmcl.2009.08.045 19720530

[B15] Khalifa S. A. M. Elias N. Farag M. A. Chen L. Saeed A. Hegazy M.-E. F. (2019). Marine natural products: a source of novel anticancer drugs. Mar. Drugs 17, 491. 10.3390/md17090491 31443597 PMC6780632

[B16] Kumar M. Gaivin R. J. Khan S. Fedorov Y. Adams D. J. Zhao W. (2023). Definition of fatty acid transport protein-2 (FATP2) structure facilitates identification of small molecule inhibitors for the treatment of diabetic complications. Int. J. Biol. Macromol. 244, 125328. 10.1016/j.ijbiomac.2023.125328 37307967 PMC10527240

[B17] Lipinski C. A. Lombardo F. Dominy B. W. Feeney P. J. (2001). Experimental and computational approaches to estimate solubility and permeability in drug discovery and development settings. Adv. Drug Deliv. Rev. 46, 3–25. 10.1016/s0169-409x(96)00423-1 11259830

[B18] McConnell O. J. Longley R. E. Koehn F. E. (1994). The discovery of marine natural products with therapeutic potential. Biotechnology 26, 109–174. 10.1016/b978-0-7506-9003-4.50011-3 7749302

[B19] Meli R. Morris G. M. Biggin P. C. (2022). Scoring functions for protein-ligand binding affinity prediction using structure-based deep learning: a review. Front. Bioinform 2, 885983. 10.3389/fbinf.2022.885983 36187180 PMC7613667

[B20] Mittal L. Kumari A. Srivastava M. Singh M. Asthana S. (2021). Identification of potential molecules against COVID-19 main protease through structure-guided virtual screening approach. J. Biomol. Struct. Dyn. 39, 3662–3680. 10.1080/07391102.2020.1768151 32396769 PMC7256355

[B21] Mittal L. Kumari A. Suri C. Bhattacharya S. Asthana S. (2020). Insights into structural dynamics of allosteric binding sites in HCV RNA-dependent RNA polymerase. J. Biomol. Struct. Dyn. 38, 1612–1625. 10.1080/07391102.2019.1614480 31057089

[B22] Padyana A. K. Gross S. Jin L. Cianchetta G. Narayanaswamy R. Wang F. (2019). Structure and inhibition mechanism of the catalytic domain of human squalene epoxidase. Nat. Commun. 10, 97. 10.1038/s41467-018-07928-x 30626872 PMC6327030

[B23] Pattar S. Adhoni S. A. Kamanavalli C. M. Kumbar S. S. (2020). *In silico* molecular docking studies and MM/GBSA analysis of coumarin-carbonodithioate hybrid derivatives divulge the anticancer potential against breast cancer. Beni-Suef Univ. J. Basic Appl. Sci. 9, 36. 10.1186/s43088-020-00059-7

[B24] Purushotham N. Singh M. Paramesha B. Kumar V. Wakode S. Banerjee S. K. (2022). Design and synthesis of amino acid derivatives of substituted benzimidazoles and pyrazoles as Sirt1 inhibitors. RSC Adv. 12, 3809–3827. 10.1039/d1ra06149f 35425455 PMC8981170

[B25] Rasheed M. A. Iqbal M. N. Saddick S. Ali I. Khan F. S. Kanwal S. (2021). Identification of lead compounds against scm (fms10) in Enterococcus faecium using computer aided drug designing. Life (Basel) 11, 77. 10.3390/life11020077 33494233 PMC7909823

[B26] Shivakumar D. Williams J. Wu Y. Damm W. Shelley J. Sherman W. (2010). Prediction of absolute solvation free energies using molecular dynamics free energy perturbation and the OPLS force field. J. Chem. Theory Comput. 6, 1509–1519. 10.1021/ct900587b 26615687

[B27] Tong J. Zhao S. (2021). Large-scale analysis of bioactive ligand conformational strain energy by *ab initio* calculation. J. Chem. Inf. Model 61, 1180–1192. 10.1021/acs.jcim.0c01197 33630603

[B28] Tong X. Wang D. Ding X. Tan X. Ren Q. Chen G. (2022). Blood-brain barrier penetration prediction enhanced by uncertainty estimation. J. Cheminform 14, 44. 10.1186/s13321-022-00619-2 35799215 PMC9264551

[B29] Tran D. P. Tada S. Yumoto A. Kitao A. Ito Y. Uzawa T. (2021). Using molecular dynamics simulations to prioritize and understand AI-generated cell penetrating peptides. Sci. Rep. 11, 10630. 10.1038/s41598-021-90245-z 34017051 PMC8137933

[B30] Wang L. Wu Y. Deng Y. Kim B. Pierce L. Krilov G. (2015). Accurate and reliable prediction of relative ligand binding potency in prospective drug discovery by way of a modern free-energy calculation protocol and force field. J. Am. Chem. Soc. 137, 2695–2703. 10.1021/ja512751q 25625324

[B31] Xiao M. Xu J. Wang W. Zhang B. Liu J. Li J. (2023). Functional significance of cholesterol metabolism in cancer: from threat to treatment. Exp. Mol. Med. 55, 1982–1995. 10.1038/s12276-023-01079-w 37653037 PMC10545798

[B32] Xiong G. Wu Z. Yi J. Fu L. Yang Z. Hsieh C. (2021). ADMETlab 2.0: an integrated online platform for accurate and comprehensive predictions of ADMET properties. Nucleic Acids Res. 49, W5–W14. 10.1093/nar/gkab255 33893803 PMC8262709

[B33] Xu R. Song J. Ruze R. Chen Y. Yin X. Wang C. (2023). SQLE promotes pancreatic cancer growth by attenuating ER stress and activating lipid rafts-regulated Src/PI3K/Akt signaling pathway. Cell Death Dis. 14, 497. 10.1038/s41419-023-05987-7 37542052 PMC10403582

[B34] You W. Ke J. Chen Y. Cai Z. Huang Z.-P. Hu P. (2022). SQLE, A key enzyme in cholesterol metabolism, correlates with tumor immune infiltration and immunotherapy outcome of pancreatic adenocarcinoma. Front. Immunol. 13, 864244. 10.3389/fimmu.2022.864244 35720314 PMC9204319

